# The Mitochondrial Cytochrome Oxidase Subunit I Gene Occurs on a Minichromosome with Extensive Heteroplasmy in Two Species of Chewing Lice, *Geomydoecus aurei* and *Thomomydoecus minor*

**DOI:** 10.1371/journal.pone.0162248

**Published:** 2016-09-02

**Authors:** Lucas L. Pietan, Theresa A. Spradling, James W. Demastes

**Affiliations:** Department of Biology, University of Northern Iowa, Cedar Falls, Iowa, United States of America; University of the Sunshine Coast, AUSTRALIA

## Abstract

In animals, mitochondrial DNA (mtDNA) typically occurs as a single circular chromosome with 13 protein-coding genes and 22 tRNA genes. The various species of lice examined previously, however, have shown mitochondrial genome rearrangements with a range of chromosome sizes and numbers. Our research demonstrates that the mitochondrial genomes of two species of chewing lice found on pocket gophers, *Geomydoecus aurei* and *Thomomydoecus minor*, are fragmented with the 1,536 base-pair (bp) cytochrome-oxidase subunit I (*cox1*) gene occurring as the only protein-coding gene on a 1,916–1,964 bp minicircular chromosome in the two species, respectively. The *cox1* gene of *T*. *minor* begins with an atypical start codon, while that of *G*. *aurei* does not. Components of the non-protein coding sequence of *G*. *aurei* and *T*. *minor* include a tRNA (isoleucine) gene, inverted repeat sequences consistent with origins of replication, and an additional non-coding region that is smaller than the non-coding sequence of other lice with such fragmented mitochondrial genomes. Sequences of *cox1* minichromosome clones for each species reveal extensive length and sequence heteroplasmy in both coding and noncoding regions. The highly variable non-gene regions of *G*. *aurei* and *T*. *minor* have little sequence similarity with one another except for a 19-bp region of phylogenetically conserved sequence with unknown function.

## Introduction

Mitochondrial DNA analysis has proven an exceptionally useful tool for population genetics, intraspecific phylogeography, and systematics [1, 2]. Among bilaterian animals, there is a high degree of conservation in the number and composition of mitochondrial protein-coding genes, although there is variability the arrangement of genes and in the number and composition of tRNA genes [3]. Because mitochondrial genes are evolutionarily conserved in such a straightforward manner among animals, homologous mitochondrial genes can be used to recover and compare evolutionary histories and molecular evolutionary patterns between disparate organisms such as vertebrate hosts and their insect parasites. For example, the mitochondrial cytochrome-oxidase subunit I gene (*cox1*) has been used to compare the evolutionary histories of pocket gophers and their chewing lice (e.g., [4, 5]). Lice, however, are exceptional in the degree to which their genomes are rearranged from the typical circular chromosome. While most arthropods differ from one another only in tRNA composition on the single mitochondrial circle [3], species of the Psocodea (barklice, booklice, and parasitic lice) studied to date have shown remarkable variation in mtDNA genomes both in terms of gene order and chromosomal structure. The single mitochondrial chromosome that is typical of nearly all bilaterian animals is simply rearranged in some lice, but fragmented into a range of types of minicircles in other louse species, some of which are so small that they only bear a single protein-coding gene, one tRNA gene, and a varying amount of other non-coding DNA [6, 7, 8, 9, 10]. The cause of fragmentation of the mitochondrial genomes of these louse species remains unknown, although a variety of ideas have been suggested (summarized by [8]), including the apparent loss of the nuclear gene, mtSSB, associated with mtDNA replication [7].

Within the Phthiraptera (parasitic lice), mitochondrial genome rearrangements as they are currently understood can be categorized in 3 types [7]. Type 1 minicircles are found as reductions from a full-sized genome; species described with type 1 minicircles also bear the full-length “master chromosome” as a special form of heteroplasmy, or within individual sequence variation. Type 2 and type 3 minicircles, occur in the absence of a full-length master chromosome. These genome types are differentiated by the number of protein-coding genes on each minicircle, ranging from “several full length major genes” in type 2 minicircles to a single protein-coding gene with a relatively large amount of non-coding sequence per chromosome in type 3 minicircles.

The type 3 minicircle that bears the *cox1* gene in human body- and human head-lice (*Pediculus humanus* and *P*. *capitis*) shows substantial heteroplasmy in both the coding and non-coding portion of the chromosome [11]. Herd et al. [12] demonstrate that the rate of heterplasmy for louse *cox1* can be higher than that of human (*Homo sapiens*) or onion thrip (*Thrips tabaci*). Because nearly 7 times more within-individual sequence variation occurs in the non-coding region than in the nearly equally sized coding region of the *cox1* chromosome, Herd et al. [12] suggest heteroplasmy in those regions may predominantly be a result of recombination between mitochondrial minichromosomes rather than substitution mutations or insertion/deletion events. However, substantial variation in degree of heteroplasmy occurs among different individual human head lice and among the minichromosomes of the louse genome, leading Xiong et al. [12] to conclude that there is not a simple relationship between fragmentation of the genome and level of heteroplasmy.

The chewing lice of pocket gophers are members of the Suborder Ischnocera and Family Trichodectidae [13]. The only other trichodectid louse mitochondrial genome reported to date has at least three type-3 minicircles with the remainder of the mitochondrial genome undetermined [7]. All chewing lice of pocket gophers belong to one of two genera, *Thomomydoecus* or *Geomydoecus*, which are well-differentiated genera morphologically [14, 15] and genetically [16, 4] and likely sister lineages among the Trichodectidae [17]. These genera are frequent cohabitants that appear to partition resources on individual hosts [18].

To begin assessment of the nature of the mitochondrial genome of chewing lice of pocket gophers, we tested for the presence of a *cox1* minicircle chromosome in representatives of both *Geomydoecus* and *Thomomydoecus* using “outward-facing” (reverse-complement) versions of PCR primers that normally amplify an internal portion of the *cox1* gene in chewing lice. We cloned PCR products to allow more accurate assessment of the nucleotide sequence of these regions and to assess the level of heteroplasmy within individual chewing lice of both species.

## Materials and Methods

Pocket gophers (*Thomomys bottae*) were collected from Socorro County, New Mexico, with approval from the New Mexico Department of Game and Fish (Permit #3500) using procedures in keeping with guidelines set by the American Society of Mammalogists [19]. Macabee traps were used, which euthanized the pocket gophers upon capture. This study was approved by the University of Northern Iowa Institutional Animal Care and Use Committee. Individual lice were removed from gophers and total DNA was extracted from 2 adult *G*. *aurei* (voucher numbers 39.6.J and 1379.16) and 1 adult *T*. *minor* (voucher number Th3243.1-A) using the DNeasy Blood &Tissue Kit (QIAGEN). Manufacturer’s recommendations were followed with the following exceptions: Prior to DNA extraction, individual louse bodies were placed on a freezer block under a dissecting microscope and punctured twice using a #2 insect pin. Carrier RNA was added to AL Buffer before addition of ethanol. Incubation before elution was increased to 3 minutes, and DNA from each louse was eluted in 30 μl AE buffer. Following DNA extraction, cleared louse bodies were mounted on microscope slides for preservation as vouchers.

To determine the nucleotide sequence of the coding sequence of *cox1*, polymerase chain reaction (PCR) was used to amplify a 1,054 base-pair (bp) region of the gene with primers LCO1490 [20] and COI-2all (referred to as H7005 in [4]) for individuals 1379.16 and Th3243.1-A (*G*. *aurei* and *T*. *minor*, respectively). All reactions were run with a negative control to ensure products were free of contamination. All reactions contained 1 μl DNA (approximately 0.5 ng) and GoTaq Clear Hot Start Master Mix (Promega, Madison, WI) in a 10.0 μl reaction. Thermal cycles included: 2 minutes at 95°C, then 40 cycles of 45 seconds at 94°C, 45 seconds at 45°C, 45 seconds at 72°C, followed by 10 minutes at 72°C. The PCR product for *G*. *aurei* was cut out of an agarose gel and re-amplified under the same conditions. PCR products were purified using ExoSap-IT (USB, Cleveland, OH) and sequenced with the amplification primers. Sequences were screened for error and edited manually using Geneious 8.1.7 [21].

To test the possibility that the *cox1* gene exists on a small, circular minichromosome and to assess heteroplasmy, two new primers were created to amplify DNA of both *G*. *aurei* individuals (39.6J and 1379.16) and *T*. *minor* (Th3243.1-A) in an outward direction (away from each other) from within the *cox1* gene: GCaurie80 (5’-CTACCAGATATTTACCCGCCTC-3’) and aurei1019 (5’GGCGGGATAACAGGTTTGGTT-3’). Thermal cycles were: 1 minute at 94°C, then 35 cycles of 45 seconds at 94°C, 45 seconds at 48°C, and 90 seconds at 72°C, followed by 10 minutes at 72°C. PCR products were cloned using the TOPO TA Cloning kit and the pCR 4-TOPO vector with Invitrogen Top 10 *Escherichia coli* competent cells (Life Technologies, Carlsbad, CA). Bacteria were spread on LB agar x-gal ampicillin plates (TEKnova, Hollister, CA) and allowed to grow over night at 37°C. Colonies that appeared to contain DNA inserts were picked at random and PCR was done to amplify the inserted DNA using either M13F or T7 forward primers with the reverse primer, M13R. PCR conditions were: 2 minutes at 95°C, 30 cycles of 45 seconds at 94°C, 45 seconds at 52°C, and 90 seconds at 72°C, followed by ten minutes at 72°C. DNA inserts were sequenced using R-1 and U primers.

Clone sequences were edited manually, and consensus sequences of forward and reverse reactions were created and aligned to those of other clones using Geneious 8.1.7 [21]. Consensus sequences of clones from the same individual louse were compared to assess heteroplasmy. By combining the cloned and directly sequenced regions of the *cox1* minichromosome, a full *cox1* minichromosome could be constructed for *G*. *aurei* (voucher number 1379.16) and *T*. *minor* (voucher number Th3243.1-A). Sequences for the full *cox1* minichromosome of both species and for clones (26 *G*. *aurei* and 13 *T*.*minor*) were submitted to Genbank under accession numbers KX228412—KX228451.

For completely reconstructed minichromosome sequences, the programs MITOS (with default parameters; [22]) and tRNAscan-SE (with parameters set to Source: Mito/Chloroplast, Genetic Code for tRNA Isotype Prediction: Invertebrate Mito, Cove score cutoff: 0.1; [23]) were used to find putative tRNA gene sequences. The einverted search program of EMBOSS explorer [24] was used to find DNA inverted repeats that could represent the origins of replication on the *G*. *aurei* and *T*.*minor cox1* chromosome. Parameters were set with gap penalty at 5, minimum score threshold at 15, match score at 3, and mismatch score at -4.

Full circle sequences for *G*. *aurei* and *T*. *minor* were compared using a MUSCLE alignment [25] via the plugin for Geneious 8.1.7 [21]. Percent identity values comparing DNA sequences and amino acid sequences were calculated in Geneious.

## Results

### *Geomydoecus aurei* genome organization and heteroplasmy

**Fig 1:**
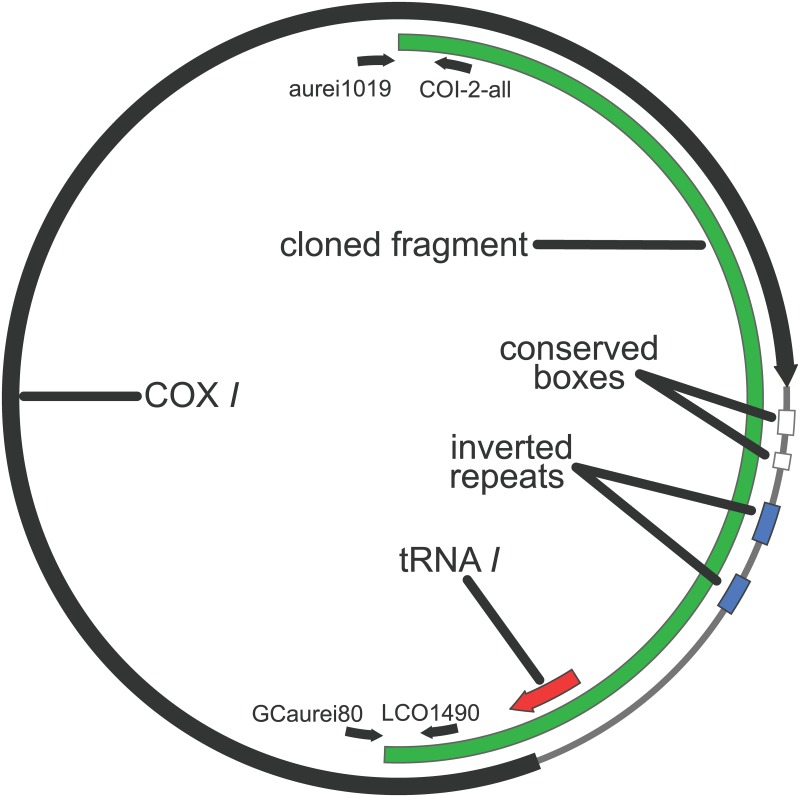
*Geomydoecus aurei cox1* Minichromosome. Fully constructed minichromosome showing the relative positions of the *cox1* gene, trn*I*, inverted repeat regions, PCR primers (shown as small black arrows), and the cloned region of the chromosome.

Amplification of the *G*. *aurei cox1* gene using primers oriented “outward” from the interior of the gene indicated that the gene occurs on a small circular chromosome (Fig 1) with only a single protein-coding region (*cox1*) on it. There was no evidence of a larger amplification product that would be expected if the *cox1* gene also occurs on a larger mitochondrial chromosome in addition to the small one (S1 Fig). Together with the rest of the *cox1* gene sequence, the full minicircle of *G*. *aurei* was 1,914 bp. The *cox1* gene was 1,536 bp long, running from a standard methionine start codon to a stop codon of TAG. MITOS and tRNAscan-SE each indicated one tRNA (isoleucine; *trnI*) of 68 bp, 6 bp of which overlapped the start of the *cox1* gene. EMBOSS explorer indicated an inverted repeat region of 91 bp within the remaining 316 bp of non-coding DNA on the minichromosome.

**Fig 2:**
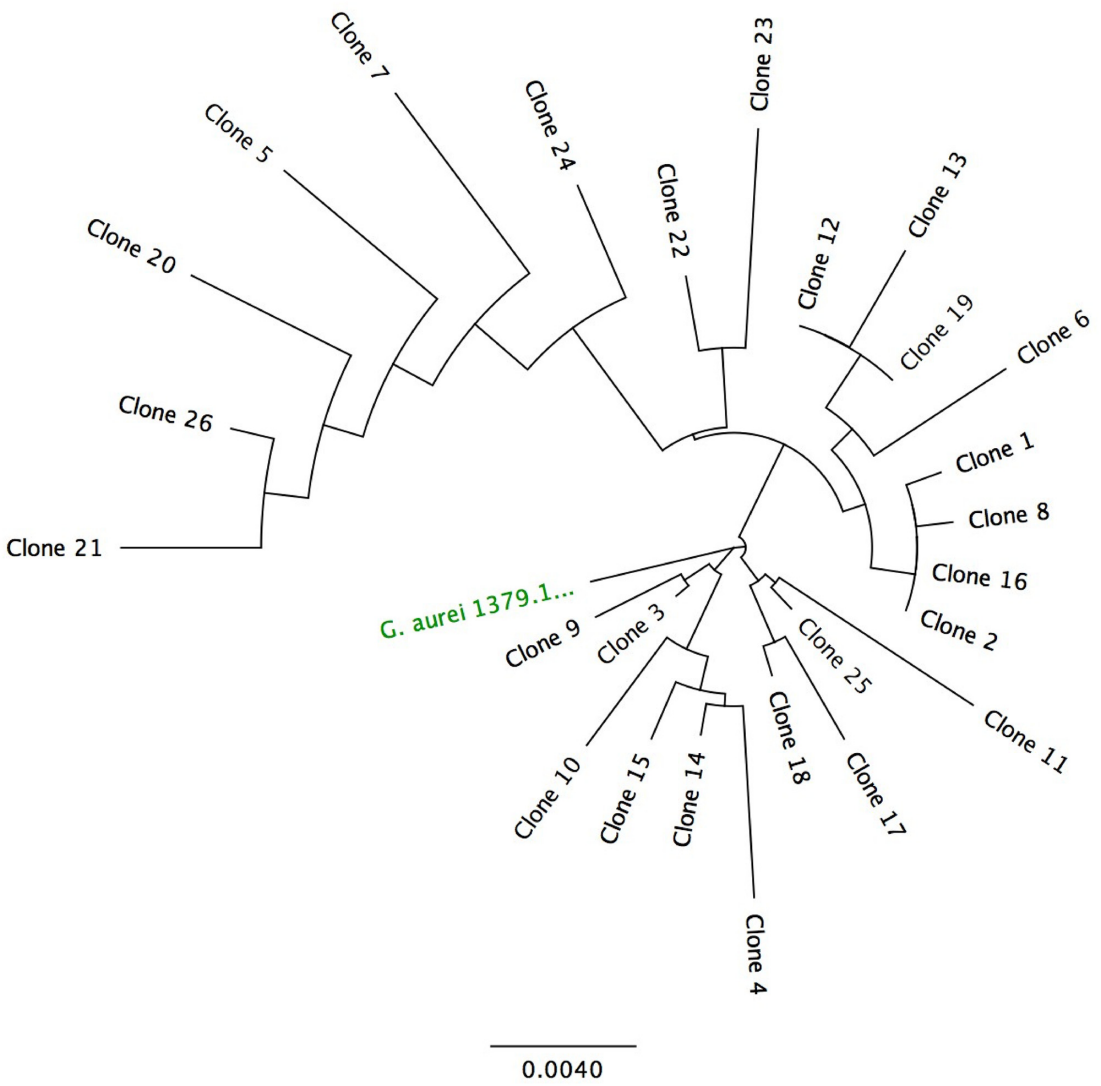
*Geomydoecus aurei* Clone Similarity. Neighbor-joining tree showing similarity between clones (numbered 1–26) derived from the DNA of a single individual of *G*. *aurei*. Sequence 1379.1 is from a different individual of the same species for comparison. Scale represents substitutions per site.

**Fig 3:**
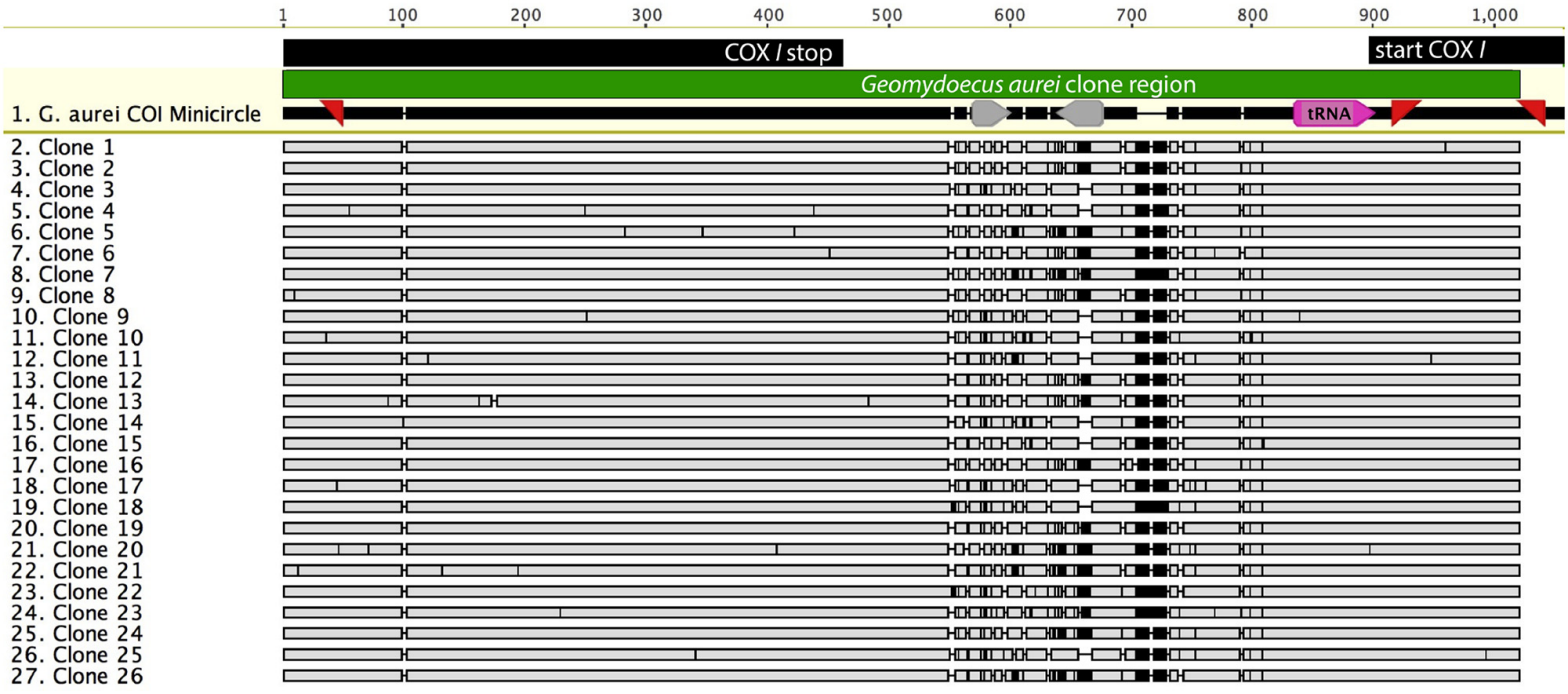
Alignment of *Geomydoecus aurei* Clones. Sequence 1: *G*. *aurei* COI minicircle is part of the full *cox1* minichromosome from louse 1379.16 in Fig 1. This sequence is from a different individual than the clone sequences below it (individual 39.6.J) and was used as a reference to map the other clone sequences. At top, black bars indicate *cox1*-coding regions, gray bars indicate inverted repeat regions, and pink indicates trn*I*. In clone sequences, vertical black marks represent differences in nucleotide composition, and horizontal bars indicate presumed insertion/deletion events in the alignment. Clone 12 and clone 19 are the only identical sequences.

Sequencing of cloned PCR products from *G*. *aurei* revealed rampant heteroplasmy, with 25 unique DNA sequences from the 26 clones analyzed (Figs 2 and 3). The non-coding regions of clones varied in length from 336 bp to 351 bp. Within the 591 bp of *cox1* sequenced for all clones, there were 26 heteroplasmic sites, 9 at the first codon position, 7 at the second, and 8 at the third (all together 0.0017 heteroplasmic sites per bp per clone). When only our first 7 clones were analyzed (for comparison with [11]), there were 8 heteroplasmic sites (0.0019 sites per bp per clone). Coding regions of two *G*. *aurei* clones were of atypical lengths, with one clone exhibiting a 1 bp deletion and the other exhibiting a 1 bp insertion, yielding frame-shift mutations. Translation of clones with normal-length coding sequences indicated unique replacement substitutions in 13 of the 26 clones, with one clone sequence coding for threonine at the start codon position. Within the tRNA region there were 2 heteroplasmic sites.

### *Thomomydoecus minor* genome organization and heteroplasmy

**Fig 4:**
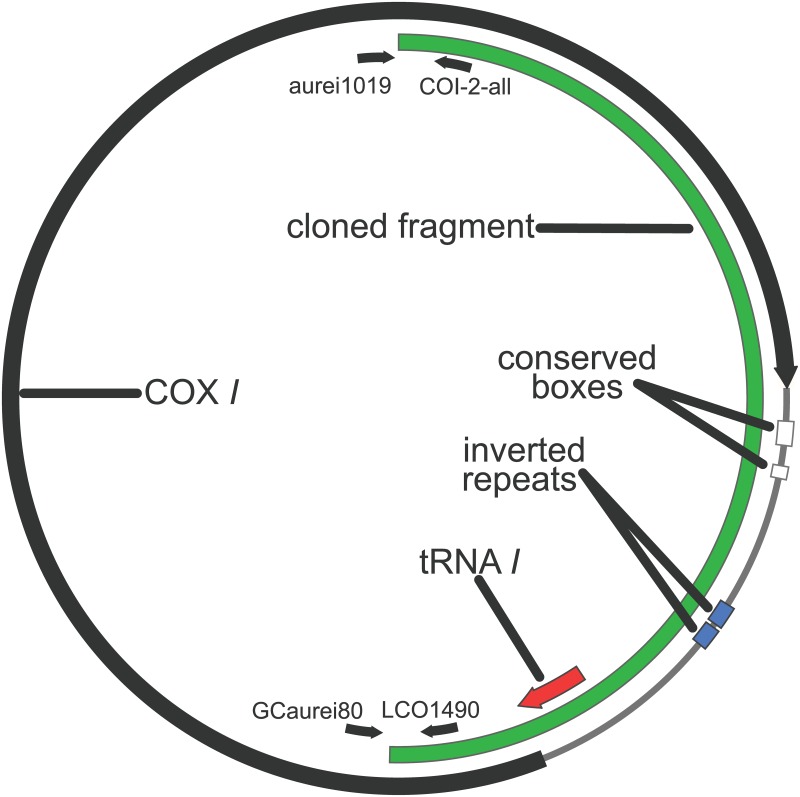
*Thomomydoecus minor cox1* Minichromosome. Fully constructed minichromosome showing the relative positions of *cox1*, trn*I*, inverted repeat regions, PCR primers (shown as small black arrows), and the cloned region of the chromosome (Clone 1).

The *T*. *minor cox1* gene also was found to occur on a minichromosome (Fig 4) 1,905 bp in size. There was no evidence of a larger amplification product that would be expected if the *cox1* gene also occurs on a larger mitochondrial chromosome in addition to the small one (S1 Fig). The minichromosome amplified had a single coding region (the *cox1* gene) of 1,536 or 1,537 bp, running either from a cysteine start codon or a non-standard 4-bp start codon region to a stop codon of TAA. MITOS and tRNAscan-SE each indicated *trnI* was 65 bp on the *T*. *minor* minichromosome with either a 3 or a 4 bp overlap with the beginning of the *cox1* gene, depending on where the start is presumed to be. An additional non-coding region of 307 bp was observed on the *T*. *minor* minichromosome, within which EMBOSS explorer identified an inverted sequence repeat of 38 bp.

**Fig 5:**
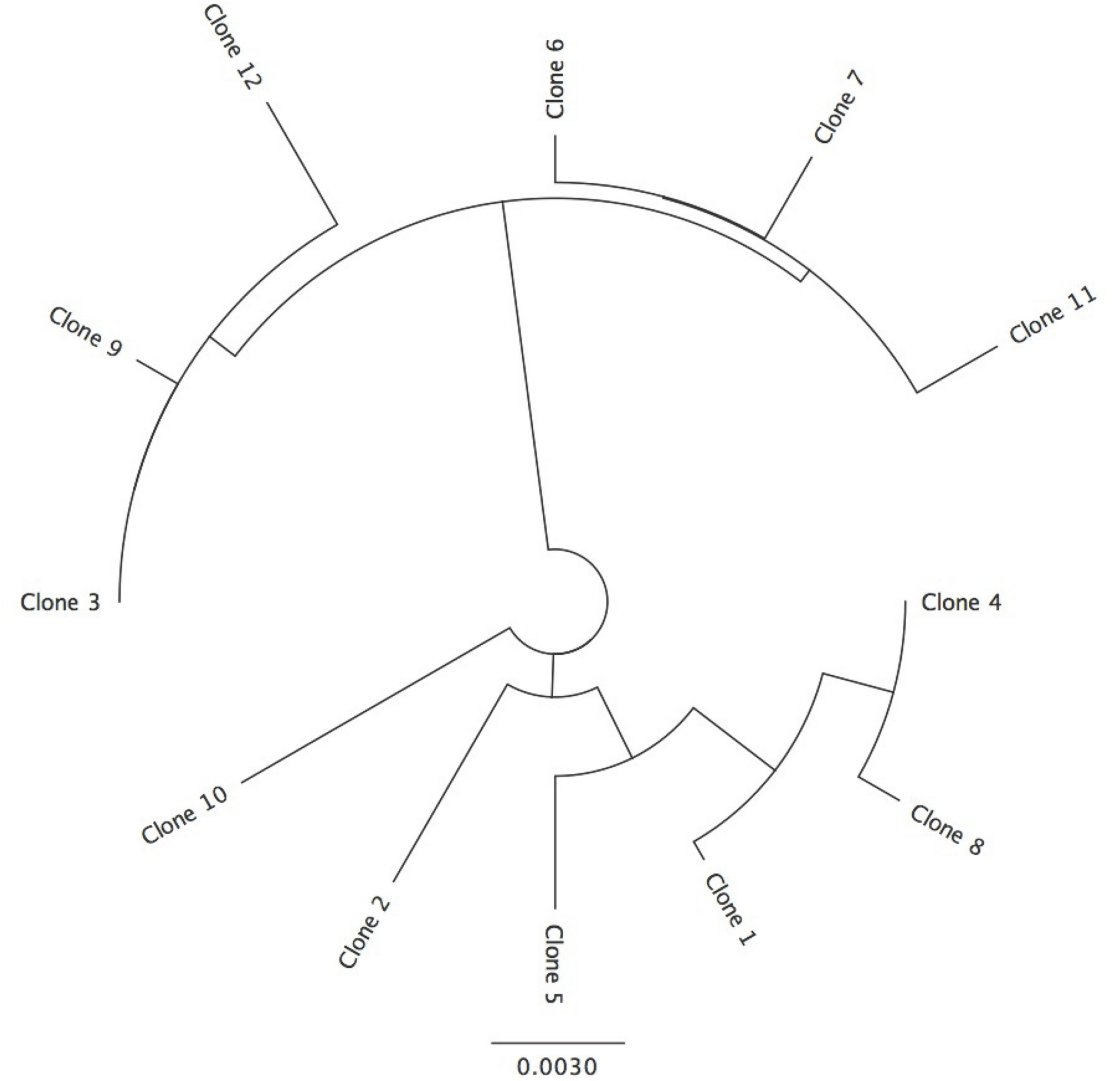
*Thomomydoecus minor* Clone Similarity. Neighbor-joining tree showing similarity between clones (numbered 1–12) derived from the DNA of a single individual of *T*. *minor*. Scale represents substitutions per site.

**Fig 6:**
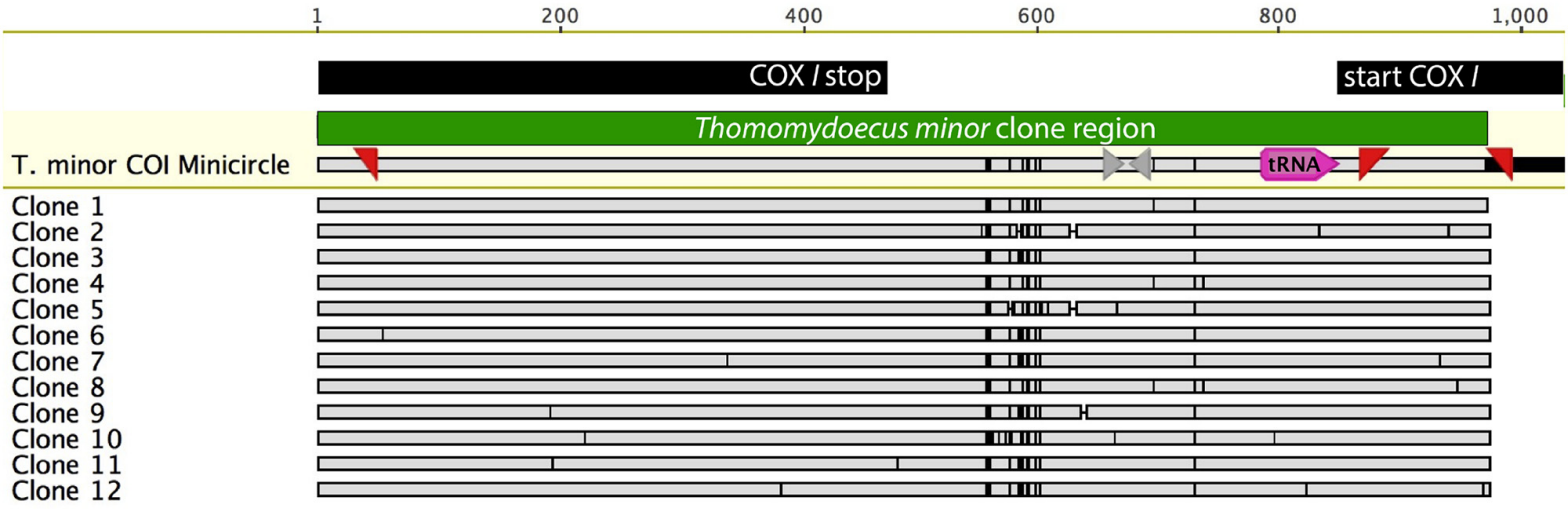
Alignment of *Thomomydoecus minor* Clones. Sequence 1: *T*. *minor* COI minicircle is part of the full *cox1* minichromosome from louse Th3243.1-A in Fig 4; this sequence is from Clone 1. At top, black bars indicate the *cox1-*coding region, gray arrows indicate inverted repeats, and pink indicates trn*I*. In clone sequences, vertical black bars represent differences in nucleotide composition, and horizontal bars indicate presumed insertion/deletion events in the alignment. All clones are unique.

Sequencing of cloned PCR products from *T*. *minor* also indicated extensive heteroplasmy. Of 12 clones sequenced, 12 unique sequences were obtained (Figs 5 and 6). The non-coding regions of these clones varied in length from 341 bp to 365 bp. Within the coding regions of the clones, there were 10 heteroplasmic sites, 4 at the first codon position, 3 at the second, and 3 at the third (0.0014 heteroplasmic sites per base pair per clone). Translation of clone sequences indicated 7 unique replacement substitutions in 6 amino acid positions of the 12 clones. Within the tRNA there were 3 heteroplasmic sites.

### Comparative Genomics

The translated sequence of the *T*. *minor cox1* gene was identical to that of *G*. *aurei* for 437 of 512 amino acids (85%), and DNA sequences for the two species were easily aligned with no gaps and 76% identity between sequences. The tRNA gene (*trnI*) of the two species was located in the same relative position on the chromosome and was within 3 bp of the same size (70 bp in *G*. *aurei* and 73 bp in *T*. *minor*), but optimum alignment of the two sequences involved 10 bp of inserted or deleted nucleotides spread over 3 separate regions (i.e., a minimum of 3 independent insertion and/or deletion events). Within the *trnI* gene, which was found overlapping the beginning of *cox1* for both species, there were stretches of 10 and 16 bp that were identical between species. The remainder of the non-protein coding region of the chromosome of the two species had little in common with the exception of a 19-bp region of sequence that was identical in the two species and identical among all clones of both species, with EMBOSS explorer finding part of that region to have inverted repeats. This sequence followed 19 and 24 bp after the stop codon of *cox1* for *G*. *aurei* and *T*. *minor*, respectively (shown as the larger of the “conserved boxes” in Figs 1 and 4). This was the second longest stretch of sequence on the *cox1* minicircle that was identical between the two species, with the only other portion of phylogenetically conserved sequence longer than this being a 23 bp region of identical sequence in the *cox1* gene. An additional 11 bp of phylogenetically conserved sequence identical between *G*. *aurei* and *T*. *minor* and identical among all clones of both individuals followed 15 bp downstream of the 19-bp phylogenetically conserved region (Figs 1 and 4). For the remainder of non-gene sequence of *G*. *aurei* and *T*. *minor*, alignment was problematic; MUSCLE alignment could generate an alignment with 54% identity between the non-protein coding regions of the two species. The inverted repeat regions of the two species were in different locations in their relative alignment, although they appeared roughly midway through the noncoding region of the chromosome for both species.

## Discussion

### *Cox1* minichromosome organization

Our PCR amplification and sequencing strategy demonstrated that the *cox1* gene of both species of lice studied here, *G*. *aurei* and *T*. *minor*, exists on a type 3 [7] minichromosome, with a single protein-coding gene on the circle, a single tRNA gene (*trnI*), and a relatively large, potentially complex noncoding region. Because we saw no evidence of larger amplification products in our work, we have no reason to suspect existence of a larger mitochondrial chromosome also containing *cox1*. Therefore, the structure of the *cox1* minicircle in chewing lice from pocket gophers is very similar to the *cox1* minicircle of another chewing louse of the family Trichodectidae, *Damalinia meyer* [7], which is found on roe deer [26]. Trichodectid lice belong to the suborder Ischnocera. While a type-3 *cox1* minicircle chromosome is not found in all members of the louse suborder Ischnocera studied to date [27], the association of *cox1* with *trnI* seen in *G*. *aurei* and *T*. *minor* appears to be a synapomorphy shared by most members of the suborder whether their mitochondrial genome is fragmented into minichromosomes or not [7]. Only 2 other minicircle sequences of *Damalinia* have been reported in the literature, so the number of chromosomes making up the remainder of that genome is unknown. Because such a wide range of possible gene arrangements are seen within the Phthiraptera, and indeed within the Ischnoceran chewing lice, a more detailed exploration of the complete mitochondrial genome of each of these trichodectid species should prove interesting and could reveal an organizational pattern unique among the Psocodea.

Cameron et al. ([7], p. 10) noted the unusually large sizes of the noncoding regions of type 3 minicircles, saying “many additional mt genomes from this group will need to be investigated to determine if there truly is a trend toward size increases in the non-coding regions of lice with type 3 minicurcular mt genomes and what, if any, function these bloated non-coding regions serve in genome maintenance, replication, or transcription”. For the *cox1* minicircle of members of the suborder Anoplura, the noncoding region is slightly larger than the *cox1* gene in human body louse [11] and greater bandicoot-rat louse [9] and nearly as long as the *cox1* gene in human head louse [11]. In the suborder Rhynchophthirina, the elephant louse has a noncoding region slightly larger than the *cox1* coding region [10]. In the suborder Ischnocera, *Damalinia meyeri* has a smaller, but still substantial noncoding region nearly half or more the size (694–1098 bp) of the *cox1* gene [7]. The lice of pocket gophers, *G*. *aurei* and *T*. *minor*, have comparatively smaller noncoding regions for the *cox1* minicircle (336–351 bp and 341–365 bp, respectively), yet the noncoding region still makes up almost 20% of the chromosome. About 1/3 of this non-protein coding sequence appears to be functional between the tRNA gene, the 19-bp and 11-bp phylogenetically conserved sequence near the end of the gene, and the inverted repeat sequences that may represent the origin of replication. Thus, available data remain insufficient for determining whether there may be a trend toward size increases considered by Cameron et al. [7], but it is becoming increasingly clear that a range of variation in noncoding sequence length occurs in lice with type 3 mitochondrial minichromosomes.

Three regions of DNA sequence that are conserved among observed mitochondrial chromosomes have been identified in *Damalinia meyeri* (“conserved sequence blocks”, [7]); whether a similar situation exists for *G*. *aurei* and *T*. *minor* cannot be determined without sequencing more of the mitochondrial genome. The noncoding regions of *G*. *aurei* and *T*. *minor*, however, are different enough from that of *Damalinia* that we could not align the sequences, and the noncoding regions of *G*. *aurei* and *T*. *minor* were quite different from one another despite their similarity in length. Therefore, if such conserved sequence blocks are present in the chewing lice of pocket gophers, they are not phylogenetically conserved to a degree that allows us to align them for different species of the family. It is possible that the 19-bp and 11-bp regions of phylogenetically conserved non-coding sequences that follow the *cox1* gene for *G*. *aurei* and *T*. *minor* are part of an important “conserved sequence block” that may be found on other mitochondrial chromosomes of these lice.

The *T*. *minor cox1* gene sequenced here displayed both a similar length and a high degree of amino-acid homology (86%) with that of *G*. *aurei*, which suggests that it is homologous to that of *G*. *aurei* despite its unusual start-codon sequence. A lack of nucleotides corresponding to stop codons in the sequence, except at the end of the gene, suggests that the *T*. *minor cox1* gene sequence recovered here is functional. Cloning of PCR products for this species revealed no heteroplasmy at the start of the gene (Fig 6); all 12 clones sequenced had the same ATGT sequence at the beginning of the gene. This ATGT start sequence also has been observed in *cox1* gene of krill [28], and other tetranucleotide sequences potentially corresponding to initiation sites have been observed in insects for the *cox1* gene [29, 30, 31]. As has been argued for these species, initiation of the *T*. *minor cox1* gene may occur employing one of two alternative atypical mechanisms. First, the *T*. *minor cox1* gene could operate with a tetranucleotide start codon ATGT, which could be edited in the resulting mRNA to remove the final uracil, thereby bringing the molecule into the reading frame to code for methionine followed by the other phylogenetically conserved amino acids of *cox1*. The mechanism for such editing is understood in detail in Trypanosomatids (reviewed in [32]; [33]). Alternatively, it is possible that the gene operates with an initiation codon yielded from the three proximal nucleotides TGT, which would code for cysteine, as has recently been proposed for a mitochondrial gene of a filarial nematode [34]. Atypical start codons for the *cox1* gene may be more common than previously suspected. Krzywinski et al. [35] provide evidence from cDNA that a mosquito species uses TCG (serine) to code for the start of *cox1*, and they argue from phylogenetic conservation of sequences in insect *cox1* that there may be a broad repertoire of codons that can serve as initiation sites for this gene.

### Heteroplasmy

Cloning revealed extensive heteroplasmy within individual *G*. *aurei* and *T*. *minor* lice. For these experiments, we used Promega GoTaq G2 Hot Start Colorless Master Mix, which includes a bacterial-derived *Taq* DNA polymerase. As such, the error rate for this enzyme should be in the range of 1.1 x 10^−4^ errors per bp or less [36, 37]. This error rate could contribute about 0.1 sequencing error in every 1,000 bp of cloned sequence for each species and would, therefore, not contribute appreciably to the heteroplasmy observed within individuals. Clearly, our cloning results have not plumbed the depths of the heteroplasmy that is present within individuals as 25 of 26 *G*. *aurei* clones and 12 of 12 *T*. *minor* clones were unique. The majority of this heteroplasmy was present in the noncoding portion of the chromosome, where purifying selection should have less effect unless it disrupts the region necessary for DNA replication or the transcription start site. The non-protein coding region may have a higher mutation rate than the coding region due to recombination with other circles bearing similar sequences [27, 11]. However, given the fact that heteroplasmy rates in some louse individuals can also be quite low, it must be the case that recombination does not always occur or that it does not always drive high rates of heteroplasmy in the noncoding regions [12]. Moreover, given that heteroplasmy can exist at a high rate even in coding regions, other mutational processes must also be important on minicircle chromosomes [12]. In our study, a substantial amount of heteroplasmy was observed within the coding region of *cox1* in *G*. *aurei* and *T*. *minor*, including sequence changes that code for amino acid substitutions within the gene and at the start codon. Such mutations could render the resulting *cox1* protein functionless. The frame-shift mutations observed in two clones of *G*. *aurei* almost certainly would render proteins derived from those chromosomes functionless. Although such an anomalous coding sequence was not recovered here, several *G*. *aurei* specimens studied in prior work in our lab were heteroplasmic with some copies of *cox1* bearing a 22 bp deletion in the middle of the gene, corresponding with a frame-shift mutation that would lead to an extremely truncated protein, providing evidence for deleterious or non-neutral mutations in the mitochondrial genome of these lice. The effects of mitochondrial gene mutations on aging in humans have been widely studied; for example, mutation in *cox1* has been shown to increase with age in human brain tissue, and this mutational burden is correlated with reduced cytochrome activity [38].

Herd et al. [11] reported much higher heteroplasmy in human head and body lice than had been observed in humans themselves or in thrips. These lice had 17 heteroplasmic sites in full COI gene for 7 clones (2.4 sites/clone) over the full coding region (1,572 bp of the gene). Because we did not clone the full length of the gene (only 591 bp of coding region), we must make comparisons on a per bp basis. Viewed this way, our observed 0.0017 and 0.0014 heteroplasmic sites/clone/bp, is approximately equal to that of human head and body lice (0.0015 heteroplasmic sites/clone/bp). Therefore, high levels of heterplasmy for the *cox1* minicircle have now been observed for both anopluran sucking lice (with degree of heteroplasmy varying among individuals; [12]) and trichodectid chewing lice, both of the groups that are known to bear a type-3 *cox1* minichromosome.

The heteroplasmy in *cox1* gene sequences of *G*. *aurei* and *T*. *minor* is not extensive enough to disrupt “reading” of a clean mitochondrial sequence using standard amplification and sequencing (i.e., non-cloning) strategies that are typical for phylogenetic studies. In fact, *cox1* gene sequences of *Geomydoecus* and *Thomomydoecus* species have been useful in past phylogenetic studies [4, 5]. Heteroplasmy is, however, extensive enough to render useless the more variable noncoding sequences of the *cox1* minichromosome, which are desired for population-genetic work. Within-individual sequence variation is so great that non-cloned amplification products cannot be effectively sequenced. More importantly, even if non-coding sequences are generated for population study, within-individual sequence heteroplasmy outstrips inter-individual sequence variation, rendering useless those sequences in a population-wide context.

### Conclusions

This study provides the first evidence for the presence of a mitochondrial minichromosome for two species of chewing lice, *G*.*aurei* and *T*. *minor*, with a single protein-coding gene and a single tRNA gene on the chromosome, making it most similar to the type-3 minichromosomes seen in some other louse species, but with *G*. *aurei* and *T*. *minor* having smaller non-protein coding regions compared to other lice [7, 10]. The *cox1* gene of *T*. *minor* has the same apparent length and amino-acid sequence as that of *G*. *aurei*. However, *T*. *minor* presents with an unusual start sequence that would depend either on RNA editing to code for a methionine (ATG) start codon or that would utilize a cysteine (TGT) start codon. As in other Ischnoceran louse species, the *cox1* gene of *G*. *aurei* and *T*. *minor* is associated with *trnI*. Extensive heteroplasmy was found within these individuals in both the coding and noncoding regions of the chromosome. Further studies are needed to determine the structures of other mitochondrial chromosomes in the lice of pocket gophers and to determine if the mitochondrial chromosomes bear conserved sequence blocks common to multiple chromosomes.

## Supporting Information

S1 Fig Agarose Gel Showing Amplification Products for *Geomydoecus aurei* and *Thomomydoecus minor Cox1* Minichromosomes. Polymerase Chain Reaction products generated using the “outward-facing” *cox1* primers of this study for two species of chewing lice from pocket gophers, *G*. *aurei* (Lane B) and *T*. *minor* (Lane C). Size standard (Lane A; mid-range DNA ladder, Fisher Scientific, Pittsburg, Pennsylvania) fragment sizes are given to the left of the image. Negative control shows no contamination (Lane D).
(PDF)
