# Supplementary material for: The Mitochondrial Cytochrome Oxidase Subunit I Gene Occurs on a Minichromosome with Extensive Heteroplasmy in Two Species of Chewing Lice, Geomydoecus aurei and Thomomydoecus minor
Source: PLoS One. 2016 Sep 2;11(9):e0162248. doi: 10.1371/journal.pone.0162248 (PMC5010254; doi:10.1371/journal.pone.0162248)
Supplement: S1 Fig — Polymerase Chain Reaction products generated using the “outward-facing” cox1 primers of this study for two species of chewing lice from pocket gophers, G. aurei (Lane B) and T. minor (Lane C). Size standard (Lane A; mid-range DNA ladder, Fisher Scientific, Pittsburg, Pennsylvania) fragment sizes are given to the left of the image. Negative control shows no contamination (Lane D). (PDF) [file pone.0162248.s001.pdf]

Supporting Information

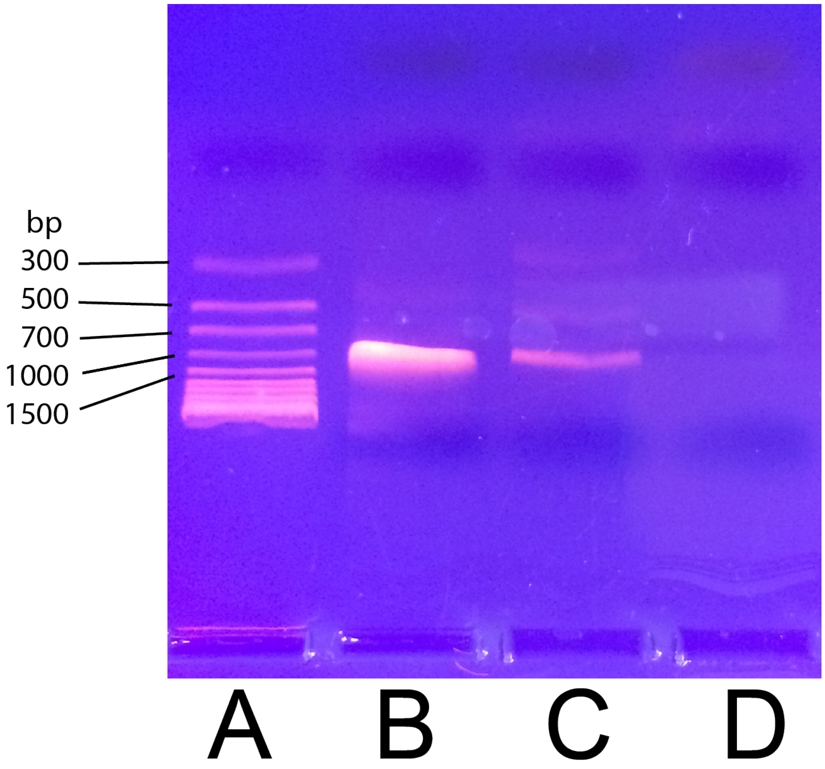

S1 Fig. Agarose Gel Showing Amplification Products for *Geomydoecus aurei* and *Thomomydoecus minor Cox1* Minichromosomes. Polymerase Chain Reaction products generated using the “outward-facing” *cox1* primers of this study for two species of chewing lice from pocket gophers, *G. aurei* (Lane B) and *T. minor* (Lane C). Size standard (Lane A; mid-range DNA ladder, Fisher Scientific, Pittsburg, Pennsylvania) fragment sizes are given to the left of the image. Negative control shows no contamination (Lane D).
